# Third-Generation Tyrosine Kinase Inhibitors Targeting Epidermal Growth Factor Receptor Mutations in Non-Small Cell Lung Cancer

**DOI:** 10.3389/fonc.2017.00113

**Published:** 2017-05-31

**Authors:** Tristan A. Barnes, Grainne M. O’Kane, Mark David Vincent, Natasha B. Leighl

**Affiliations:** ^1^Department of Medical Oncology, Princess Margaret Cancer Centre, Toronto, ON, Canada; ^2^Department of Medical Oncology, London Regional Cancer Centre, London, ON, Canada

**Keywords:** lung cancer, lung cancer treatment, epidermal growth factor receptor, tyrosine kinase inhibitors, *T790M*

## Abstract

Sensitizing mutations in the epidermal growth factor receptor (*EGFR*) predict response to *EGFR* tyrosine kinase inhibitors (TKIs) and both first- and second-generation TKIs are available as first-line treatment options in patients with advanced *EGFR*-mutant non-small cell lung cancer. Eventual resistance develops with multiple mechanisms identifiable both upon repeat biopsy and in plasma circulating tumor DNA. The *T790M* gatekeeper mutation is responsible for almost 60% of cases. A number of third-generation TKIs are in clinical development, and osimertinib has been approved by the US Food and Drug Administration for the treatment of patients with *EGFR T790M* mutant lung cancer after failure of initial *EGFR* kinase therapy. Resistance mechanisms are being identified to these novel agents, and the treatment landscape of *EGFR*-mutant lung cancer continues to evolve. The sequence of *EGFR* TKIs may change in the future and combination therapies targeting resistance appear highly promising.

## Introduction

Non-small cell lung cancer (NSCLC) accounts for over 80% of lung cancer cases and is a leading cause of morbidity and mortality internationally (1, 2). When treated with platinum-based chemotherapy, the median survival in patients with metastatic disease is 8 months (3). Mutations in the epidermal growth factor receptor gene (*EGFR*) are found in 10–15% of lung cancers in Caucasians, and 30–40% of East Asian patients (4, 5). These patients most commonly have adenocarcinoma, are lifetime non- or light smokers and are more frequently female. The most commonly described mutations are deletions in exon 19 (del19) and the exon 21 L858R point mutation (from leucine to arginine). The discovery of *EGFR* mutations as a predictor of response to tyrosine kinase inhibitors (TKIs) heralded a paradigm shift in the treatment of NSCLC (6–8).

In the advanced setting, options for first-line treatment of *EGFR*-positive lung cancer include first-generation TKIs (erlotinib, gefitinib) and afatinib, a second-generation kinase inhibitor. These agents have an impressive body of evidence confirming better response, improved progression-free survival (PFS), and quality of life compared to chemotherapy (9–16). The pooled analysis of the LUX Lung 3 and 6 studies also suggested an overall survival advantage of afatinib relative to chemotherapy in the first-line setting for the subgroup of patients with exon 19 deletions (17). Recently, afatinib has been shown to have improved PFS compared to gefitinib, however, at the expense of greater toxicity (18). Resistance to both first- and second-generation TKIs is common and develops at a median time of 9–16 months (9, 11, 14, 19). This review summarizes known mechanisms of TKI resistance, clinical approaches to resistance with a focus on third-generation *EGFR* TKIs, their preclinical and clinical evidence for use, and future directions to improve the outcomes of patients with *EGFR* mutation-positive lung cancer.

## Resistance to First- and Second-Generation Inhibitors

By performing biopsies in patients with progression on first-generation TKIs, Yu et al. elucidated the common mechanisms of resistance to first-generation TKIs (20). In approximately 60% of cases, a *T790M* point mutation in exon 20 was identified. Other mechanisms include downstream signaling pathway mutations in *BRAF* or *PIK3CA*, or parallel signaling pathway activation via changes in *MET, HER2, FGFR*, and *AXL*. A small group (3%) had histologic transformation: epithelial to mesenchymal transition or high-grade neuroendocrine transformation. A total of 18% did not have an identifiable cause. Multiple mechanisms of resistance were shown in 10% cases and have been postulated to be up to 15% of cases in other series (20–22).

More recently, the rate of *T790M* mutation have been reported to be much higher when analyzing circulating tumor DNA (ctDNA), highlighting the limitations of a single biopsy in the context of tumor heterogeneity (23). Tissue biopsies are associated with risks, delays, and an increased economic burden (24). Liquid biopsies are an attractive alternative to this and can accurately detect *T790M* mutations in ctDNA with a high positive predictive value. In the study by Oxnard et al., of 58 patients with a *T790M* negative tissue biopsy, one-third had *T790M* detected in plasma with similar response rates (RRs) to patients with the mutation identified in tumor biopsy samples (25). Recently, two studies have reported the detection of *T790M* several weeks to months prior to radiological progression, which emphasizes the potential use of serial plasma monitoring in this population (26, 27). However, plasma genotyping may still result in false negatives and it is unlikely that repeat tumor biopsies in clinic can be completely eliminated for all patients. But an approach whereby initial blood-based screening is used, followed by biopsy in only those without the mutation identified, may decrease the morbidity and delays involved in serial genomic testing.

## Managing Resistance to Initial TKI Therapy

Platinum-based chemotherapy has been considered the standard treatment upon progression for patients on initial EGFR kinase therapy; however, few patients are well enough or agree to have cytotoxic chemotherapy (28). Intercalation or combination with chemotherapy has been minimally successful with added toxicity and no consistent survival benefit (29). The IMPRESS study showed that continuing TKI therapy with chemotherapy did not provide a PFS benefit and was associated with increased toxicity (30).

For oligo progressive disease, administering local therapy and continuing the original kinase inhibitor is a common approach (31). In a small single-arm phase II study (ASPIRATION), patients with minimally symptomatic or asymptomatic progression were randomized to continue erlotinib past progression or to stop, and those continuing remained on treatment for a median of an additional 3.7 months after the initial PFS of 11 months (32).

Despite *in vitro T790M* inhibition, the second-generation TKIs have not demonstrated significant single-agent activity in *T790M* mutation positive disease. Dual inhibition of *EGFR* signaling has generated interest, with a phase II study of afatinib and cetuximab in TKI-resistant patients, demonstrating a RR of 29% in *T790M*-positive and -negative subgroups. Thus, *EGFR* pathway signaling remains an important driver of disease, with trials ongoing (33).

The most significant development in treating resistance has been through third-generation kinase inhibitors that target *T790M*.

## Third-Generation TKIs

Not only do these agents have activity in *T790M* mutant lung cancer but many have the advantage of limited wild-type inhibition, therefore, overcoming toxicities associated with first- and second-generation TKIs. WZ4002, a covalent pyrimidine *EGFR* TKI, was one of the first compounds to show *in vitro* and *in vivo EGFR T790M* inhibition with relative WT sparing (34). Several agents have now been tested in clinical trials, with osimertinib recently approved by the US Food and Drug Administration (FDA) and other regulatory agencies in patients with *EGFR T790M* mutant NSCLC post failure of first-/second-generation TKIs.

### Osimertinib (AZD9291, Previously Merelitinib)

Osimertinib is an oral, irreversible TKI that forms a covalent bond with the cysteine residue in position 797 of *EGFR* within the ATP-binding site. Osimertinib and its active metabolites also interact with a number of other kinases harboring the cysteine residue. Osimertinib is a potent inhibitor of *T790M* with little wild-type activity and shows tumor regression in murine models (35).

AURA (a phase I dose escalation study) (36) was performed in patients with *EGFR* mutation-positive advanced NSCLC with acquired resistance to TKI. No dose-limiting toxicity (DLT) was observed; the most common adverse events were diarrhea, rash, anorexia, and nausea (see Table 1). The overall RR was 51% [95% confidence interval (CI), 45–58]; higher in the *T790M* mutation-positive group than the *T790M* mutation-negative group (61 versus 21%). The median PFS was 9.6 months (95% CI, 8.3 to not reached) in *EGFR T790M-*positive patients.

**Table 1:** Toxicities of third-generation tyrosine kinase inhibitors.

Agent	Osimertinib (36–39)			Rociletinib (41, 43)	Olmutinib (46–48)	Nazartinib (50, 51)	Avitinib (55)
Study	Phase I	Phase II	Phase III	Phase I/II	Phase I/II	Phase I/II	Phase I
Number of patients	253	210	419	92	93	111	25
Response rate (RR) overall	51 (45–58)	70 (64–77)	71 (65–76)	38 (NR)	54 (NR)	47 (39–55)	NR
RR T790M Positive	61 (52–70)	As above	As above	45 (31–60)	NR	NR	NR
Overall grade 3–4 toxicity	32	34	23	NR	NR	NR	4
Rash	40 (1)	40 (1)	34 (1)	<1 (0)	39 (5)	39 (14)	20 (4)
Dry skin	20 (0)	30 (0)	23 (0)	NR	NR	28 (0)	NR
Pruritus	NR	NR	NR	NR	39 (1)	32 (0)	16 (0)
Diarrhea	47 (2)	33 (<1)	41 (1)	22 (0)	55 (0)	40 (6)	44 (0)
Loss of appetite	21 (1)	NR	18 (1)	20 (1)	NR	17 (0)	NR
Nausea	22 (<0.5)	NR	16 (1)	35 (2)	38 (0)	13 (0)	16 (0)
Fatigue	17 (1)	NR	16 (1)	24 (4)	NR	21 (NR)	NR
Dyspnea	11 (2)	<1 (<1)	4 (<1)	1.5	NR (1)	NR	12 (0)
Hyperglycemia	2 (0)	NR	NR	47 (22)	NR (0)	NR	0 (0)
QTc prolongation	NR	NR	NR	12 (5)	NR (0)	NR	8 (0)
Anemia	NR	NR	NR	NR	NR	NR (6)	NR
Stomatitis	NR	NR	NR	NR	NR	23 (NR)	NR
Muscle spasms	NR	NR	NR	11 (1)	NR	NR	NR
Dose reduction	7%	3%	NR	51%	NR	NR	NR
Discontinuation of drug	6%	5%	7%	11%	4%	NR	0

*RRs reported in % (95% confidence interval)*.

*Toxicity reported in overall rate % (grade 3–4%)*.

*NR, not reported*.

*Dyspnea, dyspnea/ILD/pneumonitis reported together*.

Updated results of the 80 mg (RP2D) cohorts from three AURA studies were presented (37), and confirm a high RR (66% in phase I study and 71% in phase II studies). Encouraging duration of response (12.5 months in pooled phase II studies) and PFS (11.0 months in same pooled analysis) was seen (37).

AURA2 is a single-arm, open-label phase II study of osimertinib 80 mg daily in *T790M* mutation positive NSCLC after first-line TKI. A total of 140 (70%; 95% CI 64–77) of 199 patients (with measurable disease) achieved an objective response. There was a disease control rate (dCR) of 92%. Toxicity was manageable with low rates of grade 3 or higher toxicity (see Table 1) (38).

AURA3 was a phase III randomized trial that assessed the efficacy and safety of osimertinib (80 mg daily) versus platinum-doublet chemotherapy after initial TKI failure in 419 patients with *T790M* mutation-positive advanced NSCLC (39). The trial demonstrated superior PFS 10.1 versus 4.4 months (HR 0.30; 95% CI 0.23–0.41; *p* < 0.001) and higher RR with osimertinib, 71%, versus chemotherapy, 31% (39). Grade 3 or 4 adverse events occurred in 23% of patients on osimertinib, compared to 47% with chemotherapy (Table 1). Quality of life results are pending.

In November 2015, osimertinib received accelerated approval by the FDA, representing rapid progress through drug development—the first AURA patient was enrolled in March 2013 and FDA accelerated approval was granted in November 2015.

### Rociletinib (CO1686)

Rociletinib is an irreversible orally delivered third-generation TKI that targets *L858R*, del19, and *T790M* mutations of *EGFR* with little WT activity. Rociletinib also modifies the C797 site through covalent binding. Tumor xenograft and transgenic models documented tumor regression in preclinical studies (40). In the phase I/dose expansion study, TIGERX, 130 patients with progression following *EGFR* TKI were enrolled but the maximum tolerated dose was not reached (41). The RR was 59% in *T790M-*positive patients; however, pooled data from this TIGER-X study and the phase II TIGER-2 was initially reported to be 30.2% (42) and later updated and published as 45% (43).

The most frequent grade 3/4 AEs, which occurred in more than 10%, included hyperglycemia and QTc prolongation (Table 1). The hyperglycemia is thought to be mediated by a metabolite of rociletinib that inhibits insulin-like growth factor-1 receptor and to a lesser extent, insulin receptor kinases. Clovis has suspended development of rociletinib and terminated enrollment in clinical trials in 2016, soon after the FDA rejected the request for accelerated approval (44).

### Olmutinib (HM61713; Formerly BI148269)

Olmutinib is an oral selective inhibitor for *EGFR* including *T790M* mutant kinases and acts by binding to a cysteine residue close to the kinase domain. Potent inhibition of representative cell lines and *in vivo* activity have been reported (45). A phase I trial of 173 patients with *EGFR*-mutant lung cancer that had failed previous TKI therapy demonstrated a favorable safety profile and promising antitumor activity (46, 47). The MTD was established as 800 mg once daily. Treatment-related adverse events occurred in 87.3% of 165 patients, mainly diarrhea, rash, skin exfoliation, nausea, pruritus, decreased appetite, and dry skin. Grade 3 or greater toxicity was 2% in the initial study report (2/93), although not in the updated results (Table 1) (46–48).

In 34 patients with centrally confirmed *T790M* tumor mutations who received olmutinib at a dose greater than 650 mg daily, the RR was 59% (10 confirmed, 10 unconfirmed partial responses) and 13 achieved disease stabilization (dCR 97%). The phase II study has been suspended early with three cases of severe skin toxicity, including two reports of toxic epidermal necrolysis (one fatal), and one case of non-fatal Stevens–Johnson syndrome. The future of the drug’s development is uncertain.

### Nazartinib (EGF816)

EGF816 is an oral, irreversible *EGFR* TKI that also forms a covalent bond with C797. Low IC_50_ values and *in vivo* activity against *L858R*–*T790M* and del19–*T790M* have been reported (49). Data from the phase I/II study of EGF816 in advanced *T790M-*positive lung cancer are now available (50, 51). Dose escalation began at 75 mg daily up to 350 mg daily for capsules and 100–225 mg daily for tablets. Diarrhea, stomatitis, rash, and pruritus were the most common toxicities (see Table 1), and the confirmed RR was 44% (56/127) and dCR was 91% (NCT02108964). Combination studies with immunotherapy are now recruiting (NCT02323126; NCT02335944; NCT02900664).

### ASP8273

ASP8273 is an *EGFR* TKI that selectively and irreversibly inhibits mutant *EGFR* kinases including T790M by the formation of a covalent bond with C797. Both *in vitro* and *in vivo* studies confirm activity in *T790M* mutant lung cancer with relative WT sparing (52). In a phase I study, doses were escalated to 500 mg but the RP2D has been deemed 300 mg, although the details of DLT and maximum tolerated dose levels are not published (53). Of 60 patients treated with ASP8273 at the 300 mg dose, there was no DLT. All patients were *EGFR* positive with 90% having a *T790M* mutation. PR was demonstrated in 16 of 45 evaluable patients; dCR was 62% (*n* = 28/45). For the 40 *T790M* mutation-positive subjects with evaluable data, 38% (15/40) had PR and dCR was 65% (26/40) (NCT02113813). The Phase III SOLAR study is underway comparing initial ASP8273 with a first-generation TKI in patients with *EGFR*-mutant lung cancer (NCT02588261) (53).

### PF06747775

PF0677775 is another oral inhibitor of *EGFR T790M* with 26-fold increased selectivity of mutant versus wild-type *EGFR*. It is currently under evaluation in a phase I/II study in patients with advanced *EGFR* mutation-positive lung cancer (del19 or L858R, *T790M* positive and negative) (NCT02349633) and early results have demonstrated activity and tolerability (54).

### Avitinib (AC0010)

Avitinib is a new, irreversible, *EGFR* mutation selective TKI being evaluated in a phase I/II clinical trial (NCT02274337). In the reported dose escalation study (55), 25 patients were treated. The most common AEs were diarrhea, rash, and pruritus. Although diarrhea and rash increased in frequency in a dose-dependent manner, the majority of them were grade 1 (Table 1). There was no drug discontinuation in all treated patients. Outcomes for two patients with T790M-positive lung cancer showed partial responses. The clinical characteristics and efficacy outcomes of the remaining patients are not reported (55).

## Special Populations

### Uncommon Mutations

The “uncommon” *EGFR* mutations represent a heterogeneous group and can account for up to 10–18% of *EGFR* mutations (56, 57). The most frequent include exon 20 insertions (exon20ins), and point mutations G719X, L861Q, and S768I. The latter three mutations may have a superior response to afatinib (58). The majority of (exon20ins) are thought resistant to *EGFR* TKIs with the exception of A763_Y764insFQEA. In a preclinical study, osimertinib demonstrated potency with a wide therapeutic window in the exon20ins studied (Y764_V765insHH, A767V769dupASV, and D770_N771insNPG) (59). More recently, it has been revealed that *EGFR* amplification may occur in a subset of exon20ins. The dual *EGFR* blockade with osimertinib and cetuximab has demonstrated significant growth inhibition in *in vivo* models (60). EGF816 has shown both *in vitro* and *in vivo* efficacy in a number of exon20ins and in a patient-derived xenograft model, 100 mg/kg dosing resulted in tumor regression of 81% (49). AP32788 has also been shown to inhibit exon20ins in BA/F3 cell lines (61). The activity of third-generation TKIs in preclinical models has led to clinical trials for exon20 insertions including the phase I/II study of AP32788 (NCT02716116).

### Brain Metastases

Approximately 30–50% of patients with NSCLC develop central nervous system (CNS) disease (62, 63). The association between *EGFR* mutation-positive NSCLC and the incidence of brain metastases is controversial with some studies suggesting an increased risk of CNS disease at diagnosis (64, 65). CNS disease can reduce survival and both brain metastases and loco regional therapies can impact neurological function and quality of life. First-generation *EGFR* TKIs have shown intracranial activity with erlotinib exhibiting higher CSF concentrations than gefitinib (66). Afatinib in patients pretreated with chemotherapy and a first-generation TKI has demonstrated a CNS dCR of 66% (67). A recent preclinical study has shown superior blood–brain barrier penetration of osimertinib compared to gefitinib, afatinib, and rociletinib (68). Sustained tumor regression in a murine brain metastases model has also been reported with osimertinib, doses of 80 mg in humans were predicted to target human brain metastases using an adaptive pharmacokinetic/pharmacodynamics model (69). AZD3759 is an innovative *EGFR* TKI developed to penetrate the blood–brain barrier but does not have *T790M* activity. BLOOM (NCT02228369) is a study testing the safety and efficacy of osimertinib 160 mg/day and AZD3759 in NSCLC patients with leptomeningeal disease; early data have reported disease control in three-quarters of patients and responses in 7 of 20 patients (70).

Osimertinib CNS activity was also confirmed in AURA and AURA2 (71). In the recently reported phase III study of osimertinib versus platinum-pemetrexed doublet, a significant improvement in PFS in patients with brain metastases was evident in the osimertinib group (8.5 versus 4.2 months; hazard ratio 0.32; 95% CI, 0.21–0.49) (39).

## Resistance to Third-Generation TKIs and Combination Treatment

### *C797S* and Other *EGFR*-Dependent Mechanisms

The point mutation *C797S* in exon 20 represents the most common resistance mechanism identified in third-generation *EGFR* TKIs. Most third-generation TKIs use the site of the cysteine amino acid located at position 797 for covalent binding, and the serine amino acid substitution reduces the capacity for TKI binding. Analysis of both plasma and tissue has confirmed this as a mechanism of resistance to osimertinib and olmutinib (72, 73). *C797S* as a cause of rociletinib resistance has been found to be much lower, but emergence of other uncommon *EGFR* mutations including *L798I, L692V*, and *E709K* have been implicated (23). Interestingly in preclinical models, if the activating mutation (del19 or *L858R*) is retained in the presence of *C797S* but without *T790M*, the tumor remains sensitive to gefitinib or afatinib. If *T790M* is present, *in vitro* analysis has demonstrated partial cetuximab sensitivity (74). Cetuximab with EAI045, a novel *EGFR* resistance mutation selective allosteric inhibitor was also effective in a mouse model of the triple-mutant *EGFR L858R/T790M/C797S* (75). Necitumumab is also being trialed in combination with osimertinib (Table 2) (NCT02496663, NCT02789345). Notably, brigatinib with or without the combination of an anti-EGFR antibody has demonstrated activity in preclinical models for the “triple-positive” tumors (76). First- and third-generation TKIs may also be combined effectively, but this is only likely if the *C797S* and *T790M* mutations occur *in trans* (77). Other acquired *EGFR* mutations have been reported by Chabon et al. including L798I, L762V, and E709K (23). *EGFR* amplification and copy number alterations are also important resistance mechanisms.

**Table 2:** Ongoing combination studies targeting resistance mechanisms.

Target	Trial name	Clinical trial identifier	Status
Epidermal growth factor receptor (EGFR)	*EGFR* inhibitor AZD9291 and necitumumab in treating patients with *EGFR*-positive stage IV or recurrent non-small cell lung cancer (NSCLC) who have progressed on a previous *EGFR* tyrosine kinase inhibitor (TKI)	NCT02496663	Recruiting
EGFR	A study of ramucirumab (LY3009806) or necitumumab (LY3012211) plus osimertinib in participants with lung cancer	NCT02789345	Recruiting
Vascular endothelial growth factor (VEGF)			
VEGF	Osimertinib and bevacizumab as treatment for *EGFR-*mutant lung cancers	NCT02803203	Recruiting
JAK1	An open-label phase 1/2 study of INCB039110 in combination with osimertinib in subjects with NSCLC	NCT02917993	Recruiting
BCL-2	Osimertinib and navitoclax in treating patients with *EGFR*-positive previously treated advanced or metastatic NSCLC	NCT02520778	Recruiting
ABL1/SRC	Dasatinib and osimertinib (AZD9291) in advanced NSCLC with *EGFR* mutations	NCT02954523	Recruiting
TORC1/2	TORC1/2 inhibitor INK128 and *EGFR* inhibitor AZD9291 in treating patients with advanced *EGFR* mutation-positive NSCLC after progression on a previous *EGFR* TKI	NCT02503722	Recruiting
MET	Study of safety and efficacy of EGF816 in combination with INC280 in NSCLC patients with *EGFR* mutation	NCT02335944	Recruiting
PD-1	Study of efficacy and safety of nivolumab in combination with EGF816 and of nivolumab in combination with INC280 in patients with previously treated NSCLC	NCT02323126	Recruiting
MET			

### RAS/RAF/MEK

Cell line studies have identified the RAS pathway as important in emerging osimertinib resistance, including mutations in NRAS as well as copy number gains. The *BRAF V600E* mutation is also a known acquired resistance mutation (78). The addition of selumetinib (MEK inhibitor) delayed and prevented resistance in preclinical models and tumor regression has been documented in an osimertinib-resistant transgenic mouse model (79). The TATTON phase 1b study is a three-arm trial of TKI naive and pretreated patients that includes combinations of osimertinib with selumetinib (AZD6094), savolitinib, a MET inhibitor, and durvalumab (NCT02143466).

### *MET* Amplification

*MET* amplification as a cause for *EGFR TKI* acquired resistance has been described in case reports and crizotinib led to a response in one of these (80–82). In a further study of rociletinib resistance, *MET* amplification accounted for 26% of patients. Patient-derived xenograft models in this study were again successfully targeted using crizotinib (83). Notable in the xenografts and in the case report by Planchard et al., selective pressure permitted the emergence of *MET* amplifications without detectable T790M suggesting that *MET* may induce resistance to third-generation TKIs. Chabon et al. also described the preexistence of co-occurring *MET* amplification with T790M, which correlated with inferior responses to rociletinib (23).

### Immunotherapy Combinations

Although immune checkpoint inhibitors have made huge advances in shifting the treatment paradigm in NSCLC, their role in *EGFR*-mutant disease is unclear. The third arm of TATTON investigated the combination of osimertinib with durvalumab and has reported early data (84). Patients were treated with osimertinib 80 mg daily with varying dosing and scheduling of durvalumab. In *EGFR* TKI naïve patients, the RR was 70% and in pretreated *T790M-*positive patients and *T790M*-negative patients RR was 67 and 21%, respectively. The combined rate of interstitial lung disease was 38% and as such this arm is currently on hold. Similarly, the CAURAL (NCT02454933) study investigating the durvalumab combination versus single-agent osimertinib has been halted due to toxicity concerns.

### Other Resistance Mechanisms

Other potentially targetable resistance mechanisms include *HER2* amplification, *FGFR1* amplification, and the *PIK3CA E545K* mutation. Epithelial to mesenchymal transition (EMT) and small cell transformation are also well recognized. Preclinical models have successfully targeted EMT with Akt inhibitors (40). Navitoclax, a BCL-2 inhibitor when combined with WZ4002, was shown to induce greater apoptosis than with the *EGFR* TKI alone (85). A phase 1b study is accruing (NCT02520778). Vascular endothelial growth factor (VEGF) and *EGFR* pathways are intimately related. The upregulation of VEGF receptors may be responsible for *EGFR* resistance and combination studies are ongoing (86) (Table 2). Early trials have already confirmed the benefit of dual inhibition with bevacizumab and erlotinib (87, 88). One small retrospective study has suggested the possibility of rechallenging with *EGFR* TKIs in addition to bevacizumab to gain further disease control (89).

## Future Directions

It is not just the complexity of resistance mechanisms that poses challenges to physicians treating the *EGFR* mutation-positive population. It is also unclear as to whether third-generation TKIs should be used in the first-line setting or should remain the option for *T790M* resistance in the second line. The results of the phase III FLAURA study which compares osimertinib to either gefitinib or erlotinib are awaited. The ADAURA study will also investigate the potential role of adjuvant osimertinib in stage IB–IIIA resected NSCLC with and without chemotherapy. The roadmap of resistance continues to grow and it is very likely that ctDNA analysis will at least complement if not replace repeat tumor biopsies in building the knowledge of resistance mechanisms. Studies have already demonstrated the emergence of *T790M* prior to radiographic changes but whether this should mean a switch in TKI is uncertain at present.

## Author Contributions

Conception and design: TB and GO. Manuscript writing; final approval of manuscript: all authors.

## Conflict of Interest Statement

TB: no conflict of interest; GO: no conflict of interest; MV: honoraria: AstraZeneca, B-I, Roche BMS, Merck, and Novartis; NL: accredited CME honoraria: AstraZeneca, BMS, MSD, and Pfizer; Institutional research: Novartis.
